# A systematic review of biodiversity and demographic change: A misinterpreted relationship?

**DOI:** 10.1007/s13280-019-01276-w

**Published:** 2019-11-23

**Authors:** Marion Mehring, Nicolai Mehlhaus, Edward Ott, Diana Hummel

**Affiliations:** 1 “Governance von Ökosystemleistungen”, Leibniz Centre for Agricultural Landscape Research (ZALF), Eberswalder Straße 84, 15374 Müncheberg, Germany; 2 ISOE – Institute for Social-Ecological Research, Biodiversity and People, Hamburger Allee 45, 60486 Frankfurt am Main, Germany; 3 Senckenberg Biodiversity and Climate Research Centre SBiK-F, Ecosystem Services and Climate, Frankfurt am Main, Germany

**Keywords:** Biodiversity, Demographic change, Driver, Human population dynamics, Millennium Ecosystem Assessment, Systematic review

## Abstract

Demographic change is supposed to be the most important indirect driver for changing biodiversity. In this article, a systematic review of 148 studies was conducted to examine the scientific evidence for this relationship and to identify potential gaps in research. We explored the spatial distribution of studies, the categories addressed with respect to biodiversity and demographic change, and the ways in which their relationships were conceptualised (spatially and temporally) and valued. The majority of studies were carried out in Africa, Europe and North America. Our analysis confirms the trend that demographic phenomena were mostly found to negatively influence biodiversity. However, a considerable number of studies also point towards impacts that were context dependent, either positive or negative under certain circumstances. In addition to that we identified significant gaps in research. In particular, there is a lack of addressing (1) other demographic aspects such as population decline, age structure or gender differences, (2) spatial variability of, e.g. human population growth, (3) long-term effects of demographic processes, and (4) the context dependency (e.g. regulations/law enforcement, type of human activities, and choice of scale or proxy). We conclude there is evidence that the relationship between biodiversity and demographic change is much more complex than expected and so far represented in research. Thus, we call for a social–ecological biodiversity research that particularly focusses on the functional relation between biodiversity and human activities, namely the different types, context, and interdependent dynamics (spatial and temporal) of this complex relation.

**Electronic supplementary material:**

The online version of this article (10.1007/s13280-019-01276-w) contains supplementary material, which is available to authorised users.

## Introduction

According to the Millennium Ecosystem Assessment, demographic change is the most important indirect driver of changing biodiversity (Millennium Ecosystem Assessment [MEA] 2005). The underlying mechanism involves an alteration of land use patterns through demographic developments (Sala et al. 2000; European Environment Agency (EEA) 2015; Newbold et al. 2015). Consequently, anthropogenic land use changes are the primary cause of changes in ecosystems and related species. So far, human population growth and urbanisation are considered the most important and dominant demographic developments. There is, however, spatial and temporal non-simultaneity of varying demographic processes, especially on different scales—global, regional, and local.

Robust knowledge about the effects of demographic change on biodiversity is scarce (Heiland et al. 2005; Röscheisen et al. 2009; Demuth 2010; Wagner et al. 2013). To date, changes in the size and density of human population and their effects on ecosystems and species diversity have mainly been discussed with a focus on their supposed negative impact (Ehrlich and Ehrlich 1981; Pimm et al. 1995; Thompson and Jones 1999; Cincotta et al. 2000; Estes et al. 2012; Newbold et al. 2015). However, it has been shown that this supposition cannot be generalised (Luck 2007a); the aggregated representation of human population dynamics cannot explain the correlations sufficiently (Liu et al. 2003; Peterson et al. 2007; Bradbury et al. 2014).

In fact, demographic change is a complex interaction of many different processes. It entails not only increasing densities and growing human populations but also shrinking populations, changes in the age and gender structure, migration movements, and socio-economic changes. All these processes can interrelate with each other to trigger various effects on biodiversity. To develop integrated solution strategies for the preservation of biodiversity, a better understanding of these effects of demographic changes on biodiversity is needed.

With this article, we seek to identify the scientific evidence for better understanding and explaining the relation between biodiversity and demographic change. Here, a special focus is placed on the ways in which complexity of demographic change is addressed and how its manifold effects on biodiversity are investigated in current research.

In this systematic review, we looked at 148 studies representing the current state of academic literature on the relation between biodiversity and demographic change.

In particular, we examined the following questions:

1. How are the studies on the relation between biodiversity and demographic change distributed geographically?
2. Which scales (spatial and temporal) of biodiversity and demographic change are examined and how do they relate to each other?
3. How is the relationship between demographic change and biodiversity?

## Materials and methods

### Search process

This systematic review is based on the methodical procedures described in Plieninger et al. (2013). We searched the databases of Web of Science and Google Scholar for relevant studies. The literature search was conducted between February 2016 and January 2017. Search criteria were not limited to a fixed time period, specific journal, or country. We looked at studies in English and German.

**Fig. 1:**
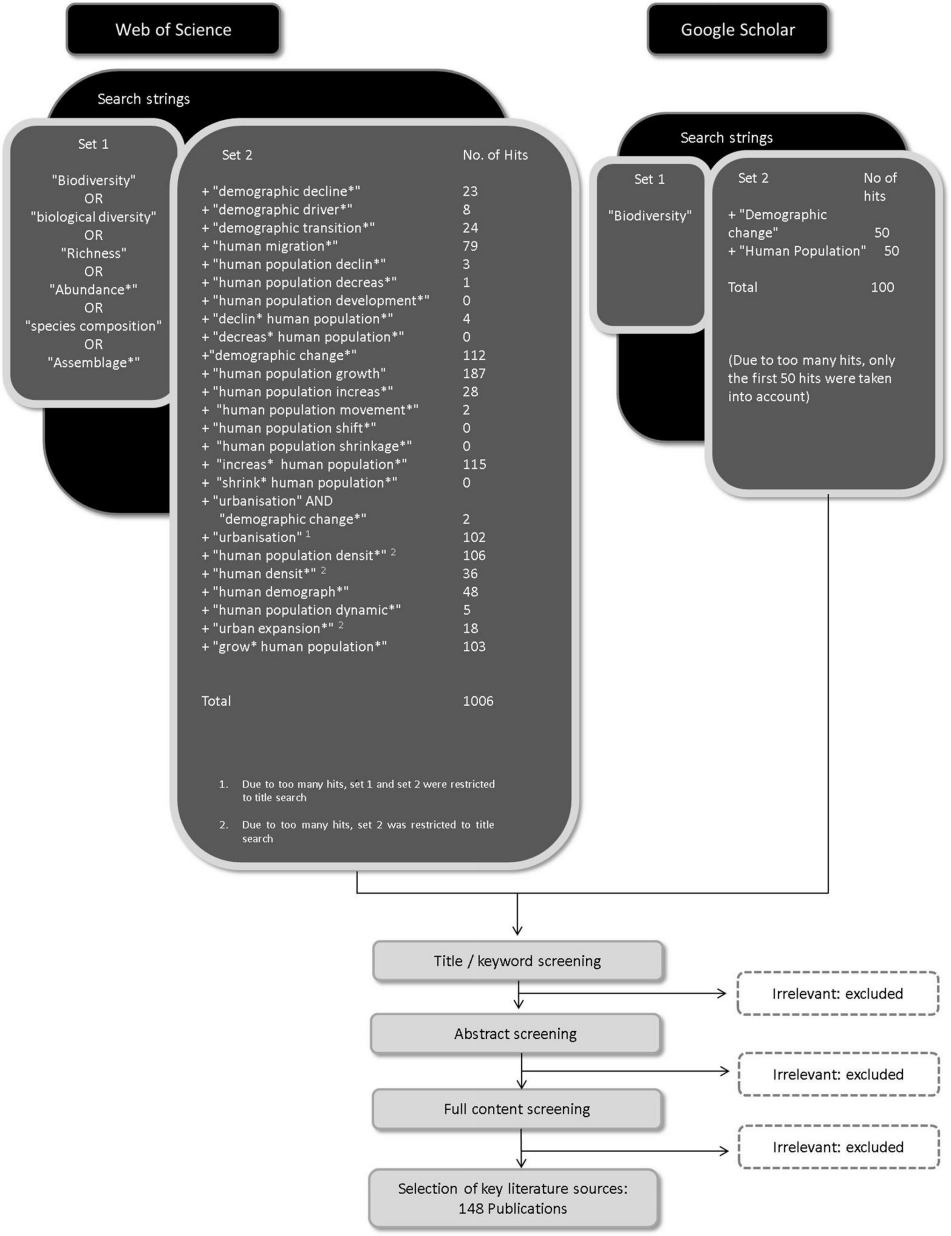
Illustration of the literature selection process

Search terms referred to aspects of demographics, demographic change, and biological diversity. Figure 1 is a schematic illustration of the search and selection process: for the search in Web of Science the basic search string represented in Set 1 (“biodiversity” OR “biological diversity” OR “richness” OR “abundance” OR “species composition” OR “assemblage”) was combined with different combinations of search terms related to demographic phenomena represented in Set 2. For all combinations of keywords, a “topic search” was conducted, except for the terms “human densit*”, “human population densit*”, and “urban expansion*”, for which Set 1 was restricted to “title search” due to too many hits per combination in topic search mode. For the combination of Set 1 with the keyword “urbanisation”, both terms were restricted to “title search”.^1^ In total, 1006 hits were obtained, which were then filtered for duplicates.

For the search in Google Scholar, a shorter search query was used, since preliminary trials with the same sophisticated approach yielded too many irrelevant results unrelated to the study focus of this analysis. We limited consideration for inclusion in the analysis to the first 50 Pdf and Word documents provided by each search. In total, 38 out of 100 hits were considered appropriate for inclusion in the further selection process. All selected studies, including titles and abstracts, were stored in a Citavi database in order to conduct a step-wise selection process.

### Selection process

The final selection of studies relevant for our analysis took place in a three-stage process adopted from Plieninger et al. (2013). Figure 1 summarises the subsequent steps in the selection process. Relevance was initially assessed from the studies’ titles. Further selection was based on the studies’ abstracts, and in a third stage, the content of the full studies was assessed. In cases of doubt, the studies were included for assessment in the next phase of the selection process. The assessment of whether a study should be considered for in-depth analysis was defined as follows: (1) the study should contain at least one keyword from the fields of biodiversity and demographic change, respectively; (2) it should include primary data collection and empirical analysis of the relationship; and (3) it should not constitute a review or meta-analysis.

After all three stages of selection, a total of 148 studies were retained for in-depth analysis. A list of the data sources can be found in Appendix S1.

### Analysis

The MAXQDA software (version 18.0) was used for final analysis of the remaining 148 studies. The software supports the qualitative evaluation of text documents. Statistical analyses can also be carried out. An analysis was conducted using a previously developed coding scheme consisting of 12 top-level categories (Table 1). Each category was assigned a definition, instructions for coding in the text, and several anchor examples to support the coding process and coders’ decision-making. The full texts were systematically analysed and corresponding text passages were marked with the appropriate code. In an iterative process, the categories were adjusted after the first 30 percent of the material had been coded. For the analysis of this article, only the categories “country/region of study”, “World Economic Situation and Prospects (WESP) country classification”, “scale of study”, “temporal scale”, “ecosystem type”, “categories of biodiversity”, “categories of demographic change”, “level of human activity”, and “direction of relation and relationship between biodiversity and demographic change” were considered.

**Table 1:** Coding system and corresponding definitions incl. codes for analysis

Category (top-level)	Definition	Codes
1.0 Publication year	Date of publication of the study	–
2.0 Country/region of study	Geographic focus of the study	–
3.0 WESP country classification	Development context of the countries under review according to the World Economic Situation and Prospects (WESP) country classification scheme	3.1 More than one country addressed; 3.2 Least developed country; 3.3 Developing country; 3.4 Developed country
4.0 Spatial scale of study	Different spatial scales of the studies	4.1 Local; 4.2 Regional; 4.3 Global
5.0 Temporal scale of study	5.1: Time scale 5.2: Analysis of temporal effect	5.1.1 Past; 5.1.2 Present; 5.1.3 Future 5.2.1 Concurrently; 5.2.2 Short-term; 5.2.3 Long-term
6.0 Habitat type	According to the MA (2005): natural/cultural landscapes or protected areas	6.1 Natural/cultural landscapes; 6.2 Protected areas
7.0 Ecosystem type	Ecosystem type classification according to the MA (2005)	7.1 Coastal; 7.2 Dryland; 7.3 Forest; 7.4 Inland Water; 7.5 Island; 7.6 Marine; 7.7 More than one ecosystem; 7.8 Mountain; 7.9 Urban/rural
8.0 Categories of biodiversity	According to the CBD definition of biodiversity: genetic diversity^a^, species diversity and habitat	8.1 Species; 8.2 Habitat
9.0 Categories of demographic change	Different aspects of demographic change are addressed in the studies	9.1 Population Density; 9.2 Population Growth; 9.3 Population Decrease; 9.4 Gender; 9.5 Age; 9.6 Migration; 9.7 Socio-Economy
10.0 Level of human activity	Different levels of human activity that were used in the studies to conceptualise demographic change, e.g. human population density as individuals per area, or villages per area	10.1 Unspecified; 10.2 Individual; 10.3 Household; 10.4 Village; 10.5 Housing; 10.6 Other
11.0 Direction of relation	Direction of impact of the relationship between demographic change and biodiversity	11.1 Unspecified; 11.2 Both; 11.3 Demographic change influences biodiversity; 11.4 Biodiversity influences demographic change
12.0 Relationship biodiversity and demographic change	Evaluation of the respective relationship between demographic change and biodiversity	12.1 Unclear; 12.2 Negative; 12.3 Context dependent; 12.4 Positive; 12.5 No effect

^a^None of the analysed studies addressed the category of genetic diversity; therefore, it was taken out of the coding scheme

The authors used the categories “unclear”, “negative”, “context dependent”, “positive”, and “no effect” to describe the potential effects. “Unclear” refers to instances when the assessed study did not specifically mention the effect of the demography–biodiversity relationship described. In these cases, it was concluded that the causal relationship could be explained by a third variable. Instances where the impact was clearly described as detrimental were labelled “negative”. Studies with a clear message that demographic phenomena exerted a favourable impact on biodiversity, or vice versa, were labelled “positive”. “No effect” refers to instances when the assessed study stated that there were no measurable effects in the demography–biodiversity relationship. “Context dependent” indicates that depending on the circumstances (e.g. type of law enforcement or kind of human activity), differing effects (positive or negative) are recorded.

## Results

### Geographical distribution of studies

**Fig. 2:**
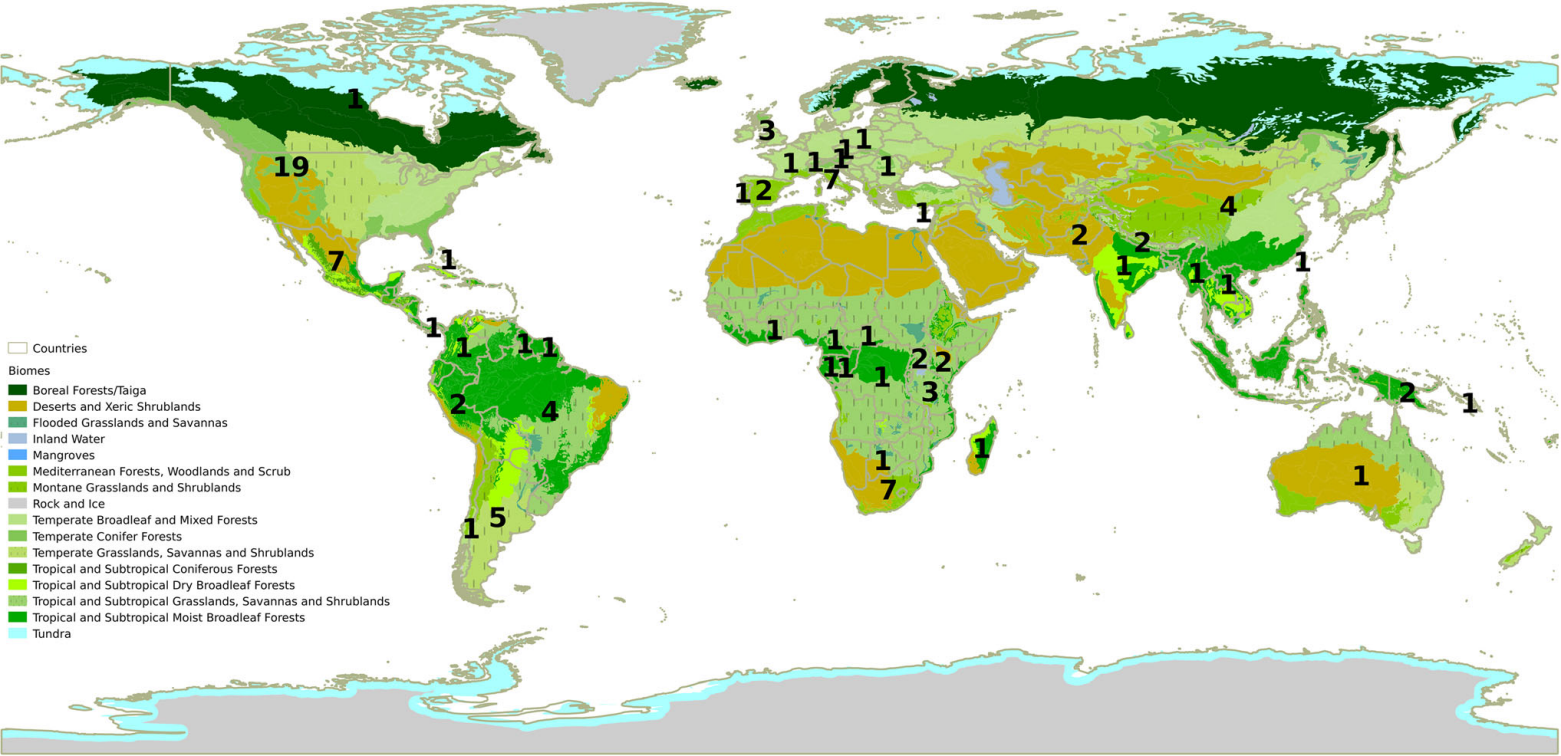
Global map of the different WWF ecoregions (2004) and the distribution of studies per country: 102 out of 148 studies. Studies on a global scale (*n *= 15) and on a continental scale (*n *= 16), and studies that looked at several countries (*n *= 7) or multiple ecoregions within one country (*n *= 8) were excluded from the map. The visualised ecoregions on the map are based on the 14 major habitat types within the WWF Ecoregions classification first published in 2001 and revised in 2004. Source: http://maps.tnc.org/gis_data.html. Accessed 14 April 2016

Figure 2 shows the studies for analysis in relation to the 14 major ecoregions worldwide. It can clearly be seen that the general distribution of studies is relatively even across Africa (*n* = 22), Europe (*n* = 20) and North American (*n* = 29), with a slight majority conducted in North America. Asia (*n* = 12), Oceania (*n* = 4), and South America (*n* = 15) are underrepresented in comparison with the other continents.

The amount of studies conducted in North America (19 in the USA alone) stands out on the map. More than two-thirds of the North American studies were assigned to the following biomes: “temperate broadleaf and mixed forests” (*n* = 11), “tropical and subtropical moist broadleaf forests” (*n* = 4), and “deserts and xeric shrublands” (*n *= 4). However, no studies were assigned to the ecoregion of “boreal forests”, which comprise the entire northern part of the continent.

In Africa, a total of 22 studies were carried out in 12 countries, with a majority of seven studies in South Africa. Considering that large parts of Africa are covered with tropical and subtropical grasslands, savannas and shrublands, and flooded grasslands, it is worth noting that these ecoregions are clearly underrepresented in the current studies. In addition, the entire northern part of Africa belongs to the ecoregion “deserts and xeric shrublands”, where no studies were conducted at all. More than half (*n* = 13) of the African studies were conducted in the equatorial region spanning the entire continent and covering mostly “tropical and subtropical moist broadleaf forests” (*n* = 9), “tropical and subtropical grasslands, savannas and shrublands” (*n* = 3), and “flooded grasslands and savannas” (*n* = 1).

In Europe, a total of 20 studies were conducted in eleven countries. Most studies were carried out in Italy (*n* = 7) and a specific biome (Mediterranean forests, woodlands and scrub). It is striking to note that no studies were conducted in Scandinavia in the boreal forests and that the temperate zones of Europe are underrepresented in comparison with the Mediterranean ecoregions.

In South America, a total of 15 studies were conducted, covering seven countries, particularly in the tropical and subtropical regions of South America. Argentina (*n* = 5) and Brazil (*n* = 4) dominate the representation of the continent (see Fig. 2). Two-thirds of these studies were conducted in regions covered by the biomes “tropical and subtropical moist broadleaf forests” (*n* = 6) and “montane grasslands and shrublands” (*n* = 4).

A limited total of 12 studies in seven countries in Asia demonstrate the continent’s underrepresentation in the corpus. Regions such as Southeast Asia, Central Asia, the Korean peninsula and Japan did not feature in the studies analysed. The most frequently investigated biomes were “tropical and subtropical moist broadleaf forests” (*n* = 4) and “temperate conifer forests” (*n* = 3).

The Oceania region was covered by four studies mainly in “tropical and subtropical moist broadleaf forests”.

In summary, the most investigated biomes in all regions of the world were “tropical and subtropical moist and broadleaf forests” (*n* = 26), “Mediterranean forests, woodlands and scrub” (*n* = 19), and “temperate broadleaf and mixed forests” (*n* = 17). Not one study covered the boreal forests and taiga regions, the world’s largest biome after the oceans. Other important biomes, which were either totally neglected or else only sparsely covered, were “tropical and subtropical coniferous forests”, “mangroves”, “temperate grasslands, savannas and shrublands”, and “flooded grasslands and savannas”. Countries such as the USA, South Africa, Italy, and Mexico dominated the amount of studies and their geographical distribution. The corpus lacked regions associated with high biodiversity, such as Southeast Asia, the western coastlines of South America, New Zealand, and Japan.

**Fig. 3:**
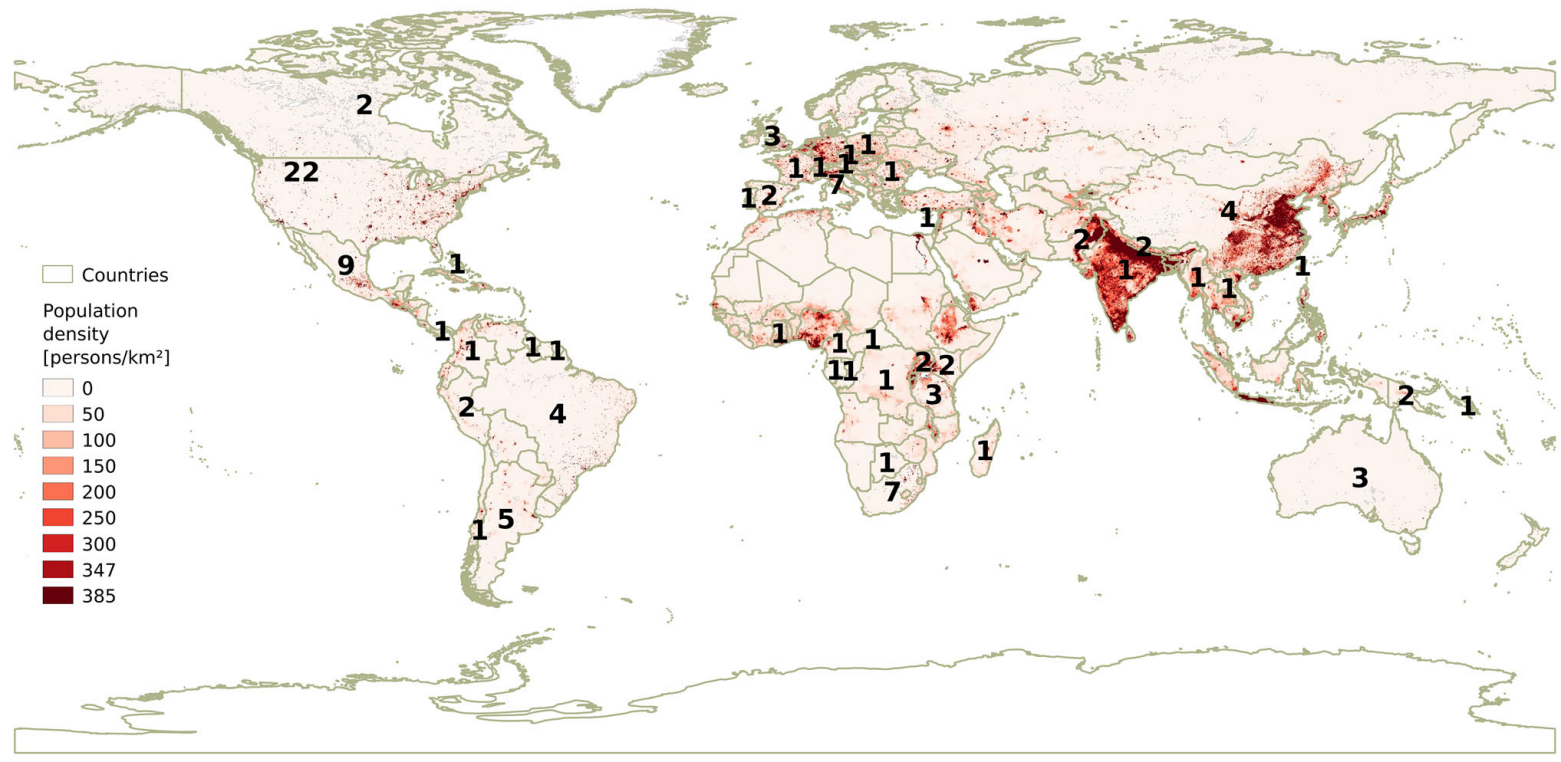
Global map showing human population densities per country and the distribution of studies per country: 110 studies out of 148 studies. Broad-scale studies on a global scale (*n *= 15) and on a continental scale (*n *= 16), and studies that looked at several countries (*n *= 7) were excluded from the map (Population Density Adjusted to Match 2015 Revision UN WPP Country Totals). Source: http://dx.doi.org/10.7927/H4HX19NJ. Accessed 30 November 2017

Figure 3 illustrates the number of studies analysed and the human population density in the regions where they were conducted.

Conspicuously, Asia and Europe featured a relatively small number of studies in areas with very high human population density. The majority of studies in South America took place in rural regions with relatively low human population density. The most differentiated distribution of studies can be seen in Africa and North America.

It was particularly striking that no studies materialised in Southeast Asia, a region rich in biodiversity with human population densities between 300 and 385 people/km^2^. Similarly, disconcerting was the likewise sparse consideration given to Central Asia and the whole Himalaya region, also known as biodiversity hotspots overlapping with high human population densities. The studies analysed in the equatorial regions of Africa (Tanzania, Uganda and Kenya) coincide with densely populated regions that are also considered biodiversity hotspots. The northern coastline of the continent was not represented in the data, even though the coastal regions of Morocco, Algeria, and Tunisia are very biodiverse regions, with human population densities of 250 people/km^2^ and above. The whole southern coastline of West Africa is considered an area of species diversity and richness that also shows high human population densities between 250 and 385 people/km^2^. However, our data showed only one study conducted in that region (Ghana). In Europe, the distribution of the assessed studies does not coincide with the most densely populated regions such as Germany or the Benelux States. The European studies clearly focused on the biodiverse regions of Spain, Portugal, and Italy. The majority of studies in South America were conducted in rural regions with rather low human population density. The studies conducted in Peru, Chile, Colombia, North-West Argentina, and Central Brazil, in particular, coincided with biodiversity hotspots. In North America, most studies coincide with areas of high population density such as the USA and Mexico.

### Scales of demographic change and biodiversity

#### Spatial aspects

A majority of 69% was conducted at the local level (studies within one country), followed by 18% regional (more than one country and/or a continent) and 13% global (ecosystems or biomes in different parts of the world) level.

**Fig. 4:**
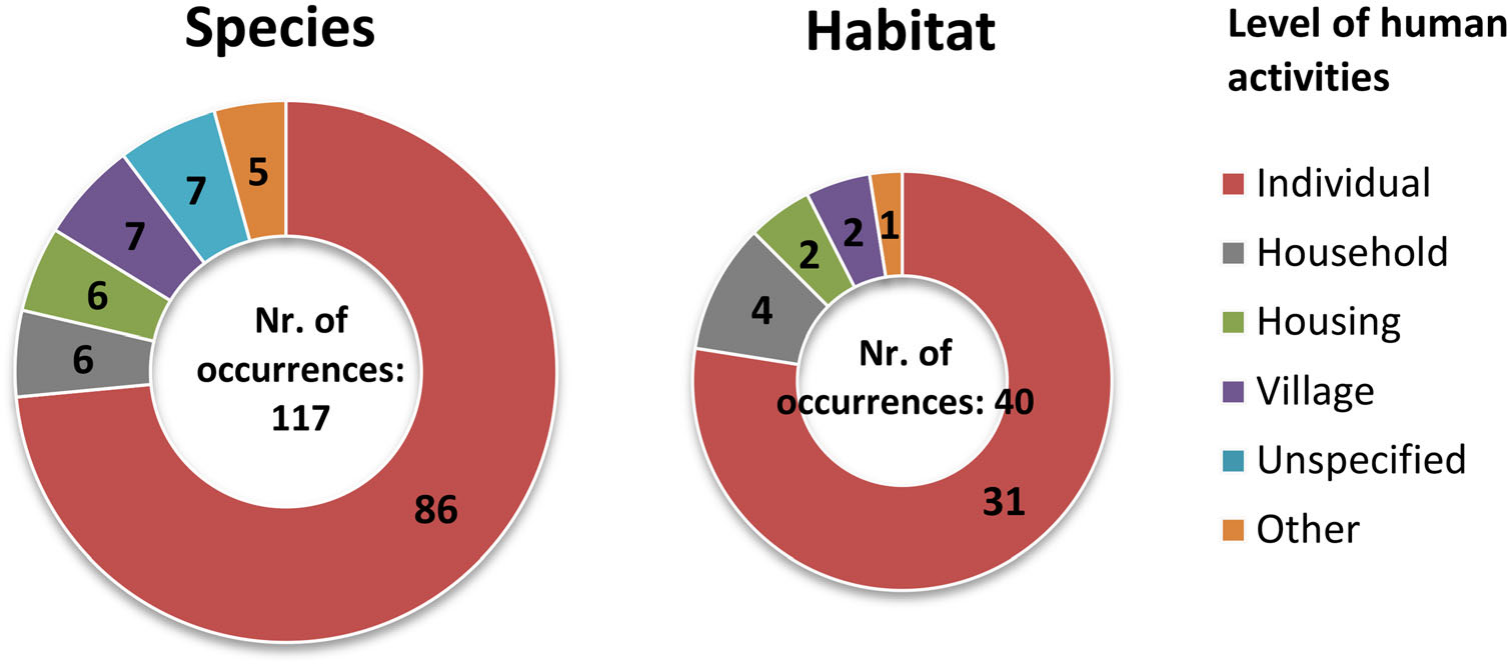
Level of human activities related to categories of biodiversity, namely habitat, species, and genetic diversity. The latter was not addressed in the studies. The numbers indicate the occurrences in the studies. The total number of occurrences exceeds the total number of studies (*n *= 148), since one study may refer to more than one level of human activity

Figure 4 shows the scales of human activity investigated in the individual studies in relation to species diversity and habitat. The level at which human activity takes place in relation to biodiversity change is important to consider, since, for example the size and composition of a household have a vital effect on per capita consumption.

It is worth noting that the studies analysed dealt more often with human activities in relation to species diversity than in relation to habitat diversity. No studies addressed the genetic diversity of species. For both biodiversity categories, most studies examined human activities at an individual level: analysis of eighty-six of the 117 occurrences of species diversity factored in human activity at this level, as did analysis of 31 of the 40 occurrences of habitat diversity. Human population density, mostly measured in individuals/km^2^, indicated individual activity.

Analysis of about one-third of the occurrences of species and habitat diversity referred to aggregated activity levels, for example based on households, housing density, or villages. Household-level activity designated activities based on the decisions made by the individuals living in one household. Housing-level activity designated the density of buildings in relation to a specific area (e.g. houses/km^2^). Village-level activity designated the number of rural settlements (e.g. Altrichter and Boaglio 2004) and the number of human communities (e.g. Ramos et al. 2014) in a specified area.

Unspecified levels of human activity only occurred in studies that investigated species diversity. The “unspecified” code was only assigned to studies that did not clearly indicate the level of human activity on which they were focusing (e.g. people/km^2^, households/km^2^, houses/km^2^). Human activities that were, for example measured on the basis of physicochemical variables (e.g. Aarif et al. 2014) or the amount of bush meat harvested (e.g. Prado et al. 2012; Cawthorn and Hoffman 2015) were coded under the “other” category.

**Fig. 5:**
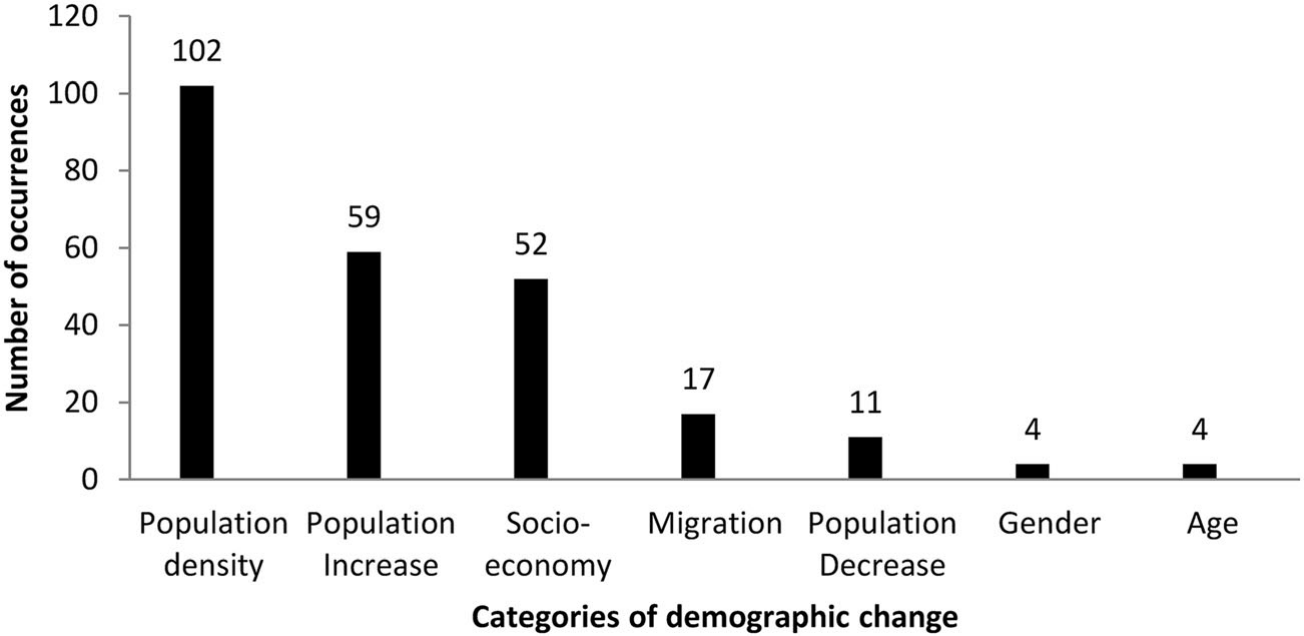
Total number of occurrences per category of demographic change addressed. The numbers indicate the occurrences in the different studies towards specific demographic processes. The total number of occurrences exceeds the total number of studies (*n *= 148), since one study may refer to more than one demographic process

The main determinants of demographic change are mortality, fertility, and migration, which themselves are influenced by a broad range of economic, social, and cultural factors. Figure 5 captures the most important processes of demographic change assessed by the studies, such as human population density, population growth and decline, and migration. It further lists gender, age, and socio-economic aspects as other important influencing factors covered. For example, a region’s socio-economic developments (e.g. poverty, sanitation, access to electricity and clean water) and the age structure of a society have a strong influence on birth rates, migration patterns, and mortality.

From our data, it is noticeable that the majority of the assessed studies focused on human population dynamics represented by human population density, since analysis of 102 out of 249 occurrences referred to human population density. Fifty-nine occurrences were related to the growth rate of human populations, which made this the second most important factor covered within the studies, followed by socio-economic aspects (*n* = 52). Several studies point out the importance of human population density and growth rates in relation to changes in biodiversity (Cincotta et al. 2000; Paradis 2018). At the same time, Cincotta et al. (2000) point out that human population variables are insufficient proxies to assess risk to biodiversity. Despite their importance, only a small number of occurrences were examined in terms of gender, age, migration, or human population decline.

However, often, the assessment of this relationship only focuses on spatial co-occurrences between biodiversity- rich regions and centres of high human population density in order to make predictions about biodiversity (Chown et al. 2003; Barbosa et al. 2010). Another study displaying the complex spatial relationship between demographic factors and biodiversity was conducted by Paradis (2018) in Southeast Asia. The author assessed the geographical distribution of terrestrial vertebrate biodiversity (mammals, birds, reptiles, and amphibians) in Southeast Asia in relation to increasing human populations and human population density. He tested for the hypothesis that the human population growth between 1990 and 2000 resulted in increased threats to biodiversity. The results, however, illustrate a differentiated spatial dimension of the demography–biodiversity relationship: species diversity increased with human population density up to about 10 people/km^2^. With human population density increasing to about 200 people/km^2^, species diversity would decrease and stabilise thereafter in open landscapes. He generally found a non-linear relationship between human population density and biodiversity in forests and open landscapes, which contradicts previous studies (Luck et al. 2010).

#### Temporal aspects

The conceptualization of time lines differed considerably amongst the different studies. Table 2 depicts the temporal aspects, namely the time scale of data collection (past, present, future) as well as the analysis of the timeline between cause and effect (concurrently, short-term, or long-term).

**Table 2:** Timeline conceptualization within the studies (n = 148)

	Category	Number of studies
Time scale (as of the year of data collection)	Past	21
	Present	120
	Future	7
Analysis of timeline between cause and effect relationship	Concurrently	93
	Short-term (< 10 years)	12
	Long-term (> 10 years)	43

Our results show that in general most of the studies (*n* = 120) focused on present research (the year of data collection is the same as those for analysis). Only a few (*n* = 21) addressed the past (data for analysis was before the year of data collection), and a minority investigated future scenarios (*n* = 7). Regarding the timeline for the cause–effect relationship, most of the studies (*n* = 93) addressed concurrently cause–effect relationships such as statistical correlations between species and human activities. Only 12 studies investigated the role of demographic processes in the past (< 10 years) and their effect on biodiversity nowadays. In total, 43 studies focussed on long-term effects (> 10 years). Of these, the largest group makes up studies addressing cause–effect relationship in the past over a long period (> 10 years). Research is lacking on long-term effects of demographic changes in the past more than 10 years ago leading to effects on biodiversity nowadays. If then, these studies refer to the topic of land abandonment (Angelstam et al. 2003; Acha and Newing 2015).

### Relationship between demographic change and biodiversity

**Fig. 6:**
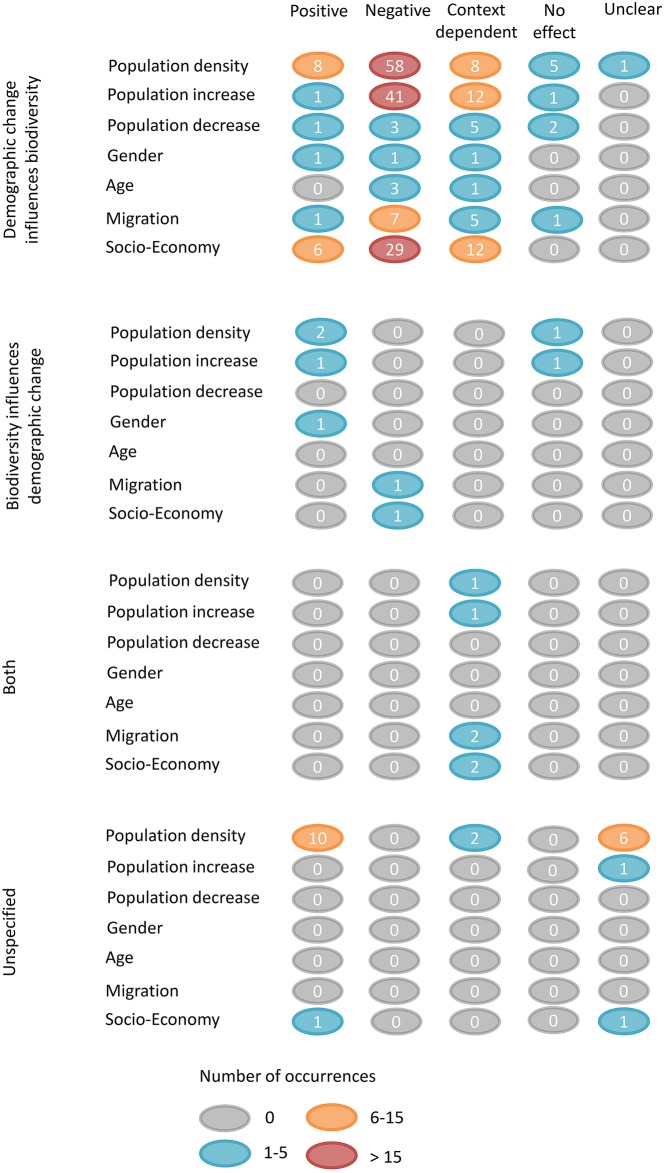
Overview of the occurrences according to the relationship between demographic change and biodiversity showing the categories of demographic change addressed. The numbers indicate the occurrences in all studies related to different demography–biodiversity relationships. The figure illustrates four manifestations of the demography–biodiversity relationship addressed in the respective studies: demographic change influences biodiversity, biodiversity influences demographic change, both (studies that referred to both directions of the potential impact), and unspecified (studies that either did not clearly reveal relational characteristics between demographic change and biodiversity or revealed a third explanatory variable)

Figure 6 provides an overview of the critical relationship between biodiversity and demographic change. In particular, it depicts the different manifestations of the biodiversity–demography relationship, i.e. the impact of demographic change on biodiversity and vice versa, and whether the assessed impact was negative, positive, context dependent, unclear or with no effect. In addition, Fig. 6 differentiates between the respective demographic processes as described above, including their impact.

Generally speaking, most occurrences indicated a negative impact of demographic change on biodiversity. The most relevant demographic processes were human population density and increase as well as socio-economy.

The majority of occurrences focused on the influence of demographic change on biodiversity. To describe this category, the authors coded instances where there were clear indications within the assessed study of whether demographic developments influence biodiversity. A very good illustration of how demographic factors can influence biodiversity on the habitat level is the deterioration of traditional cork oak landscapes in Spain due to human migration to urban centres (e.g. Acha and Newing 2015).

The second most frequently assigned occurrences fell into the “unspecified” category. The studies in question found correlations between demographic change and biodiversity but questioned the causality of the linkage. The majority of these studies conclude that the correlation is explained by a third variable. A good example of such a study is the work of Luck et al. (2010), in which six hypotheses were tested that could explain the positive correlation between human population density and bird species richness in Australia. In this case, net primary productivity is identified as one of the main factors driving spatial congruence between biodiversity and densely populated areas.

There were, however, a noteworthy number of occurrences that pointed towards a context-dependent effect that could be either positive or negative under certain circumstances. This result suggests that the relationship between demographic change and biodiversity is more complex than most current studies anticipate. In general, three different aspects of context dependency can be differentiated: First, matter of scale or choice of the proxy for both, biodiversity and demography. Some studies revealed different effects depending on analysing, e.g. abundance or diversity (Bloch and Klingbeil 2016), different types of species (incl. native versus alien) (Foster et al. 2002; McKinney 2002; Wilson et al. 2007; Hugo and van Rensburg 2008; Marini et al. 2009), and different scales (local versus coarse) (Pautasso 2007; Underwood et al. 2009). For demographic change, the results diverge depending on, e.g. the type of poverty indicator (Fisher and Christopher 2006), using within household variability versus household numbers (Carter et al. 2014), adding housing units in addition to land cover (Lepczyk et al. 2008), integrating the temporal effects of demographic processes of land abandonment (Angelstam et al. 2003; Acha and Newing 2015; López-Bao et al. 2015), or integrating gender (Swierk and Madigosky 2014). Second, the type of human activities had considerably different effects. Regarding land use, it makes a difference if you practise burning or grazing for land clearance (Bamford et al. 2014). Levi et al. (2009) and Prado et al. (2012) showed that the strategy of hunting (e.g. bow hunting versus shotgun hunting) matters. Selier et al. (2016) indicated in their study that the type of human disturbance is relevant: human population density revealed negative impact for nature, while an increase in tourism resulted in higher numbers of elephants. Third, regulations and law enforcement were proven to make a difference. A study from northern Argentina revealed that market-driven soybean expansion had a more positive effect on biodiversity than governmental sponsored programs (Grau et al. 2008).

Additionally, a reasonable number of occurrences showed that demographic change, when appropriately managed, can have a positive or at least considerably less negative impact on biodiversity. For example, Jha and Bawa (2006) quantified the effects of human population growth and development on rates of deforestation. The authors showed that in the case of high human population growth rates and low human development, deforestation rates are high, but when human development is high, deforestation rates are significantly lower, despite high human population growth. In particular, the state policies play a crucial role here as low and high human development is a product of state policies. The study found that when a policy was reversed, i.e. when logging was not supported by the government, it led to an increase in forest with an increase in human development.

Finally, only a few occurrences addressed how biodiversity influences demographic processes with no clear picture of a positive or negative relation. An impressive example of this direction of the relationship was given by Brauner-Otto (2014), who links environmental conditions to fertility in rural communities in Nepal. Finally, there are a few studies who revealed no effect between the relation of demographic change and biodiversity.

In summary, our analysis shows that in most of the studies analysed, demographic processes have a negative influence on biodiversity. However, our results also reveal a considerable number of studies with positive or context-dependent effects on biodiversity. Strongly underrepresented in the data are studies on population decrease, ageing societies, or migrating human populations.

## Discussion

Demographic change and biodiversity are critically linked in a complex manner not adequately reflected in the current state of scientific research and scientific knowledge. Often, demographic change is said to be the most important indirect driver for a loss or change in biological diversity. The authors of this study are, however, convinced that the relationship between biodiversity and demographic factors is more complex than suggested by widely cited publications such as the Millennium Ecosystem Assessment (MEA 2005). Therefore, this systematic analysis aimed to collect and assess the scientific literature in the field to provide further insights into the geographical distribution of such studies worldwide, looking at the types of biological diversity and the different types of demographic change processes that were addressed.

### Geographical distribution

Our results demonstrate an uneven spatial distribution of the studies worldwide. Whole biomes, regions, and biodiversity hotspots are underrepresented or missing from the research agenda in this field. Our data illustrate that the research coverage of areas in biodiversity hotspots is surprisingly low. This ran counter to our initial expectations of finding a focus on biodiverse regions due to their paramount social, ecological, and economic importance and to the attention the concept has gained by becoming one of the principal global conservation–prioritisation paradigms (Mittermeier et al. 2011).

The recent list of hotspots contains 35 regions worldwide (Williams et al. 2011). These regions cover 17.3 percent of the Earth’s terrestrial area and are characterised by exceptional biodiversity and considerable land-cover disturbances (Myers et al. 2000; Mittermeier et al. 2004). Generally speaking, they represent areas where human settlement, biological diversity, and environmental degradation strongly coincide (Williams 2013). The distribution and geographic focus of the analysed studies suggests major research gaps for many important biodiversity hotspots in all regions and climatic zones of the world: Southeast Asia (Sundaland), southern Asia (Indo-Burma), the mountain regions of Central Asia, Japan, the Philippines, the Himalayas, the entire western coast of South America (Tropical Andes and Chilean Winter Rainfall Valdivian Forests), Central America, the Caribbean Islands, East Africa (Horn of Africa), the northern coast of Africa as part of the Mediterranean Basin, West Africa (Guinea Forests), New Zealand, Polynesia, and Micronesia (Marchese 2015). Generating scientific and practical knowledge for these regions in the light of demographic processes should become a priority within national and international research agendas, since the areas of the most biodiverse regions worldwide are the home to two billion people (Mittermeier et al. 2011), with human populations increasing at above-average growth rates (Williams 2013). Sloan et al. (2014) highlight the severe situation in the world’s biodiversity hotspots by conducting an analysis of the natural intact vegetation showing that the area covered by natural intact vegetation reached a critical level of under ten percent.

Our findings further show that vital biomes are clearly underrepresented in studies covering the relationship between biodiversity and demographic change. The boreal forests and taiga regions, as the world’s largest terrestrial biome, are a very noteworthy example of how whole ecoregions are not being covered in scientific studies assessing the dynamics between demography and biodiversity, despite their significant importance for global climate regulation (Snyder et al. 2004; Gauthier et al. 2015). One reason for the current neglect of that biome may be the perception that the boreal regions are not affected by demographic processes as much as other regions due to comparatively low human population density and difficult access. Gauthier et al. (2015) point out that the overall human impact on the whole biome may be low, but that regionally and locally, the impact due to harvesting, agricultural activities, human settlements, mining, and road construction can be considerable. Furthermore, the cumulative effects of these activities are still unclear. The review by Gauthier et al. (2015) projects a great threat to boreal forest health under the current management regimes. They therefore call for greater attention to the boreal regions in the global debate on sustainable development, biodiversity conservation, and climate change mitigation.

Other biomes that are underrepresented in literature on the impact of demographic change, such as savannas, grassland, steppes, and shrublands, are not nearly as important for global or regional climate control as the boreal forests (Snyder et al. 2004). They often reflect rather low human population densities but are under strong pressure to be converted into agricultural land or other more intensive forms of land use that pose a great threat to biodiversity in these biomes (Cremene et al. 2005; Medan et al. 2011).

These spatial gaps in the analysed studies are in line with a larger picture of the state of biodiversity research recently characterised as problematic by several authors. Tydecks et al. (2018) report systematic spatial biases in biodiversity-related research. According to the authors, research is dominated by contributions from wealthy countries, while major research deficits can be observed in regions with high biodiversity coupled with a disproportionately high share of threatened species. Di Marco et al. (2017) come to similar conclusions when observing the state of conservation science. They reveal a disconnectedness in research between scientific focus and conservation needs. Namely, 40 percent of studies have been carried out in the USA, Australia, or the UK, and only six and ten percent in South East Asia and Africa, respectively. They conclude that “global conservation science is still poorly aligned with biodiversity distribution and conservation priorities” (Di Marco et al. 2017, p. 32). Focusing on scientific research on animal biodiversity, Titley et al. (2017) point out that specific taxonomic groups can be underrepresented or overrepresented. Research is more frequently carried out in developed countries with larger economies, while tropical countries are understudied relative to temperate countries. Investigating the global distribution of Neotropical snakes, Guedes et al. (2018) demonstrate that important ecoregions are understudied, which they trace back to these regions’ inaccessibility, low investments in local research, and a relative shortage of experts to explore huge areas. Furthermore, they point out that well-sampled areas coincide with the location of the most active universities and scientific collections.

### Scales and indicators

In general, our results support the statement that demographic phenomena mostly exert a negative impact on biodiversity. However, the range of demographic phenomena and the levels of human activities assessed are very biased towards individual activities and human population densities and growth. Further studies point out the importance of human population density and growth rates in relation to changes in biodiversity (Williams 2013). However, a curtailed assessment based on human population indicators may only deliver a distorted image of the actual risk to biodiversity. Varying structural aspects such as the spatial distribution of human populations or social implications that determine consumption patterns may be inadequately addressed by research primarily focusing on human population growth, despite their potential to affect ecosystems. Regardless of the importance of human population indicators and the attention they receive in contemporary biodiversity research, they seem to be insufficient proxies to assess risk to biodiversity. Human population density, for example does not specify the spatial distribution of human populations within a single area or inform about temporal human population dynamics happening within the same region. While the human population may be growing massively in some parts of a region, it may be declining in other parts. One metropolitan area may host most of a region’s human population but only cover a fraction of a specific ecoregion, which of course does not mean that there is no impact on biodiversity in regions far away from the metropolitan centre. There is ample evidence that urban human populations’ demand for food, fibre, water, fuel, recreation, and waste disposal has the capacity to strongly alter ecosystems more than 100 km away (Luck 2007b; e.g. Puppim de Oliveira et al. 2011). In this line, studies using human development index as an indicator should reflect that this index can be a sign of economies that do not depend anymore on resources as a direct source, e.g. wood, but might take wood from less developed countries, implying that on the global scale biodiversity is still decreasing (tele-coupling effect). The same goes for deforestation rates. Although those can be locally decreasing, this might be due to the fact that developed countries simply have no own, cheap wood sources anymore since the state of the forest has already reached a critical level locally.

In addition, our results show that research is lacking on the temporal effects of demographic change, i.e. demographic processes in the past leading to effects nowadays. Those studies particularly addressing long-term effects of former demographic processes on current biodiversity found a positive impact (e.g. land abandonment). However, comparing T0, measurements are also relevant in this respect. If biodiversity was degraded a lot because of human settlement, it might happen that it increases again after some time has passed and new species are migrating into human settlements, as those find new habitats in urban areas (e.g. birds that find food and breeding grounds in private gardens or public parks). That does not necessarily mean that human settlement had solely a positive effect on biodiversity, since most likely biodiversity was depleted during earlier phases of human settlement.

Other important demographic aspects such as age, gender, and decreasing human populations are also clearly underrepresented in currents studies, which is an indication that the current knowledge base is limited to a rather narrow set of conditional factors. In order to capture the full causal breadth of the dynamic relationship between demographic processes and biodiversity, scientific approaches are needed that go beyond the mere application of standard demography indicators. This is further emphasised by our findings that demographic phenomena are not automatically the cause of biodiversity degradation. Under specific conditions, the opposite may even be the case: demographic developments may have a positive influence on biodiversity or may not cause any harm to it.

### Complex relationship between demographic change and biodiversity

The statement that “demographic change is the most important indirect driver of biodiversity loss” does not acknowledge the complex nature of the relationship between demographic change and biodiversity. The often cited statement suggests a mono-directional relationship between demographic phenomena and biodiversity, where demographic dynamics automatically exert a negative influence on biodiversity. Our findings show that the critical demography–biodiversity relationship is complex and multifaceted in its different manifestations and requires a more diversified scientific recognition. Barbosa et al. (2013), for example point out that human population density is an insufficient indicator to make predictions on biodiversity; they argue that sampling effort distorts the results of many studies. These findings were supported by Cantarello et al. (2010), who conducted a study under similar conditions.

In addition, most studies focusing on correlations between biodiversity and demographic factors could not uncover the causality behind the common co-occurrence of biodiversity and densely populated regions. One explanatory approach suggests that this co-occurrence is based on the availability of energy for both humans and biodiversity (plants and animals) (Evans et al. 2005; Luck 2007a; Cantarello et al. 2010).

## Conclusion

In this article, we have examined selected studies on the relationship between biodiversity and demographic change. In general, our analysis confirms the trend that demographic phenomena were mostly found to negatively influence biodiversity. However, a considerable number of studies also point towards impacts that were context dependent, either positive or negative under certain circumstances. A small proportion of studies referred to instances where demographic change was related to positive effects on biodiversity.

While the influence of demographic change on biodiversity has attracted a lot of scientific attention, the influence of biodiversity on demographic change has not. The same is true vice versa: there are few published examples showing that demographic change, when properly managed, may have a positive or at least considerably lesser negative effect on biodiversity. In fact, a more detailed reflection of the generally underrepresented factors age, gender, and socio-economic aspects including the temporal effects thereof revealed interesting details about the complex relationship between demographic change and biodiversity. In particular, the spatially unequal distribution of, e.g. human population density, the level (individual, household, quarter) and context of human activity (regulations, enforcement), and finally the time delay of demographic processes do matter in this case. So far, most of the studies still focus on the spatial distribution of human population activities and its impact on biodiversity, we found only a few studies addressing long-term effects of demographic change. This raises the question of whether temporal demographic processes (e.g. short- term migration or long-term changes in age structure) exert similar impacts on biodiversity over time as spatial demographic processes.

In general, our results demonstrate the importance of the complexity in the relationship between demographic change and biodiversity. The findings confirm the need for a critical perspective on the demography–biodiversity relationship in its different manifestations. It cannot be presumed that demographic change always exerts a negative impact on biodiversity. Indeed, there is evidence that demographic indicators might not be suitable measures to analyse the risk to biodiversity.

From a policy perspective, our results highlight the importance to generate scientific and practical knowledge for biodiversity hotspot regions. In the light of demographic processes, this should become a priority within national and international research agendas, since the areas of the most biodiverse regions worldwide are home to two billion people, with human populations increasing at above-average growth rates. Furthermore, none of the analysed studies addressed the role of policies in shaping demographic developments such as education or human development programmes. More robust knowledge on this topic is needed.

Our results also provide new ideas for future research. These involve the addressing of different ecoregions worldwide and demographic processes in addition to human population growth. Our findings call for a broader consideration of important mediating factors such as age, gender, and socio-economic activities to make more exact predictions for the risk to biodiversity. In addition, important biomes such as the boreal forests and taiga regions—some of the world’s largest biomes—were not represented at all in our studies. Other important biomes such as tropical and subtropical forests, mangroves, temperate grasslands, savannas, and shrublands were only sparsely considered. In addition, the general global distribution of studies is biased towards certain countries and geographical regions that neglect important biodiversity hotspots such as Southeast Asia and the western coastlines of South America.

Finally, the results clearly call for a social–ecological biodiversity research that particularly focusses on the functional relation between biodiversity and human activities, namely the different types, context, and interdependent dynamics (spatial and temporal) of this complex relation (Mehring et al. 2017).

## Electronic supplementary material

Below is the link to the electronic supplementary material.

Supplementary material 1 (PDF 94 kb)

## Footnotes

1 Note that Web of Science differentiates automatically between American and British spelling and shows results for both styles.
