# Supplementary material for: A systematic review of biodiversity and demographic change: A misinterpreted relationship?
Source: Ambio. 2019 Nov 23;49(7):1297–312. doi: 10.1007/s13280-019-01276-w (PMC7190604; doi:10.1007/s13280-019-01276-w)
Supplement: Supplementary file 1 — Supplementary material 1 (PDF 94 kb) [file 13280_2019_1276_MOESM1_ESM.pdf]

Ambio
Electronic Supplementary Material
This supplementary material has not been peer reviewed
Title: **A systematic review of biodiversity and demographic change: A misinterpreted relationship?**
Marion Mehring, Nicolai Mehlhaus, Edward Ott, Diana Hummel

Appendix S 1. Studies (148) of the systematic review

Aarif, K.M., S.B. Muzaffar, S. Babu, and P.K. Prasadan. 2014. Shorebird assemblages respond to anthropogenic stress by altering habitat use in a wetland in India. *Biodiversity and Conservation* 23: 727–740. doi: 10.1007/s10531-014-0630-9

Acha, A., and H.S. Newing. 2015. Cork oak landscapes, promised or compromised lands?: A case study of a traditional cultural landscape in southern Spain. *Human Ecology* 43: 601–611. doi: 10.1007/s10745-015-9768-7

Allen, A.P., T.R. Whittier, D.P. Larsen, P.R. Kaufmann, R.J. O'Connor, R.M. Hughes, R.S. Stemberger, S.S. Dixit, et al. 1999. Concordance of taxonomic composition patterns across multiple lake assemblages: Effects of scale, body size, and land use. *Canadian Journal of Fisheries and Aquatic Sciences* 56: 2029–2040. doi: 10.1139/cjfas-56-11-2029

Altrichter, M., and G.I. Boaglio. 2004. Distribution and relative abundance of peccaries in the Argentine Chaco: Associations with human factors. *Biological Conservation* 116: 217–225. doi: 10.1016/S0006-3207(03)00192-7

Angelstam, P., L. Boresjö-Bronge, G. Mikusiński, U. Sporrong, and A. Wästfelt. 2003. Assessing village authenticity with satellite images: a method to identify intact cultural landscapes in Europe. *Ambio* 32: 594–604 (eng).

Araújo, M.B. 2003. The coincidence of people and biodiversity in Europe. *Global Ecology and Biogeography* 12: 5–12. doi: 10.1046/j.1466-822X.2003.00314.x

Aung, M., K. Khaing Swe, T. Oo, K. Kyaw Moe, P. Leimgruber, T. Allendorf, C. Duncan, and C. Wemmer. 2004. The environmental history of Chatthin Wildlife Sanctuary, a protected area in Myanmar (Burma). *Journal of Environmental Management* 72: 205–216 (eng). doi: 10.1016/j.jenvman.2004.04.013

Bahaa-el-din, L., R. Sollmann, L.T.B. Hunter, R. Slotow, D.W. Macdonald, and P. Henschel. 2016. Effects of human land-use on Africa's only forest-dependent felid: The African golden cat *Caracal aurata*. *Biological Conservation* 199: 1–9. doi: 10.1016/j.biocon.2016.04.013

Bamford, A.J., D. Ferrol-Schulte, and J. Wathan. 2014. Human and wildlife usage of a protected area buffer zone in an area of high immigration. *Oryx* 48: 504–513. doi: 10.1017/S0030605313000215

Barbosa, A.M., D. Fontaneto, L. Marini, and M. Pautasso. 2010. Is the human population a large-scale indicator of the species richness of ground beetles? *Animal Conservation* 13: 432–441. doi: 10.1111/j.1469-1795.2010.00363.x

Bloch, C.P., and B.T. Klingbeil. 2016. Anthropogenic factors and habitat complexity influence biodiversity but wave exposure drives species turnover of a subtropical rocky inter-tidal metacommunity. *Marine Ecology* 37: 64–76. doi: 10.1111/maec.12250

Brashares, J.S., P. Arcese, and M.K. Sam. 2001. Human demography and reserve size predict wildlife extinction in West Africa. *Proceedings of the Royal Society B* 268: 2473–2478 (eng). doi: 10.1098/rspb.2001.1815

Brauner-Otto, S.R. 2014. Environmental Quality and Fertility: The effects of plant density, species richness, and plant diversity on fertility limitation. *Population and Environment* 36: 1–31 (eng). doi: 10.1007/s11111-013-0199-3

Brewer, T.D., J.E. Cinner, R. Fisher, A. Green, and S.K. Wilson. 2012. Market access, population density, and socioeconomic development explain diversity and functional group biomass of coral reef fish assemblages. *Global Environmental Change* 22: 399–406. doi: 10.1016/j.gloenvcha.2012.01.006

Buchanan, G.M., P.F. Donald, L.D.C. Fishpool, J.A. Arinaitwe, M. Balman, and P. Mayaux. 2009. An assessment of land cover and threats in important bird areas in Africa. *Bird Conservation International* 19: 49. doi: 10.1017/S0959270908007697

Buij, R., B.M. Croes, G. Gort, and J. Komdeur. 2013. The role of breeding range, diet, mobility and body size in associations of raptor communities and land-use in a West African savanna. *Biological Conservation* 166: 231–246. doi: 10.1016/j.biocon.2013.06.028

Burgess, N.D., A.J. Balmford, N.J. Cordeiro, J. Fjeldså, W. Küper, C. Rahbek, E.W. Sanderson, J.P.W. Scharlemann, et al. 2007. Correlations among species distributions, human density and human infrastructure across the high biodiversity tropical mountains of Africa. *Biological Conservation* 134: 164–177. doi: 10.1016/j.biocon.2006.08.024

Burlakova, L.E., A.Y. Karatayev, V.A. Karatayev, M.E. May, D.L. Bennett, and M.J. Cook. 2011. Biogeography and conservation of freshwater mussels (Bivalvia Unionidae) in Texas: patterns of diversity and threats. *Diversity and Distributions* 17: 393–407. doi: 10.1111/j.1472-4642.2011.00753.x

Butler, J.R.A., T. Skewes, D. Mitchell, M. Pontio, and T. Hills. 2014. Stakeholder perceptions of ecosystem service declines in Milne Bay, Papua New Guinea: Is human population a more critical driver than climate change? *Marine Policy* 46: 1–13. doi: 10.1016/j.marpol.2013.12.011

Cadet, P., E. Pate, and J. Thioulouse. 2003. Relationship of nematode communities to human demographics and environment in agricultural fields and fallow lands in Senegal. *Journal of Tropical Ecology* 19: 279–290. doi: 10.1017/S0266467403003316

Cano-Ramírez, M., B. de La Tejera, A. Casas, L. Salazar, and R. García-Barrios. 2012. Migración rural y huertos familiares en una comunidad indígena del centro de México. *Botanical Sciences* 90: 287–304 (es).

Cantarello, E., C.E. Steck, P. Fontana, D. Fontaneto, L. Marini, and M. Pautasso. 2010. A multi-scale study of Orthoptera species richness and human population size controlling for sampling effort. *Die Naturwissenschaften* 97: 265–271 (eng). doi: 10.1007/s00114-009-0636-4

Carboni, M., W. Thuiller, F. Izzi, and A. Acosta. 2010. Disentangling the relative effects of environmental versus human factors on the abundance of native and alien plant species in Mediterranean sandy shores. *Diversity and Distributions* 16: 537–546. doi: 10.1111/j.1472-4642.2010.00677.x

Cardillo, M., A. Purvis, W. Sechrest, J.L. Gittleman, J. Bielby, and G.M. Mace. 2004. Human population density and extinction risk in the world's carnivores. *PLoS Biology* 2: E197 (eng). doi: 10.1371/journal.pbio.0020197

Carter, N.H., A. Viña, V. Hull, W.J. McConnell, W. Axinn, D. Ghimire, and J. Liu. 2014. Coupled human and natural systems approach to wildlife research and conservation. *Ecology and Society* 19: 43. doi: 10.5751/ES-06881-190343

Cawthorn, D.-M., and L.C. Hoffman. 2015. The bushmeat and food security nexus: A global account of the contributions, conundrums and ethical collisions. *Food Research International* 76: 906–925. doi: 10.1016/j.foodres.2015.03.025

Cayuela, L., J.M.R. Benayas, and C. Echeverría. 2006. Clearance and fragmentation of tropical montane forests in the highlands of Chiapas, Mexico (1975-2000). *Forest Ecology and Management* 226: 208–218. doi: 10.1016/j.foreco.2006.01.047

Chown, S.L., B.J. van Rensburg, K.J. Gaston, A.S.L. Rodrigues, and A.S. van Jaarsveld. 2003. Energy, species richness, and human population size: conservation implications at a national scale. *Ecological Applications* 13: 1233–1241. doi: 10.1890/02-5105

Cincotta, R.P., J. Wisnewski, and R. Engelman. 2000. Human population in the biodiversity hotspots. *Nature* 404: 990–992 (eng). doi: 10.1038/35010105

Concepción, E.D., M.K. Obrist, M. Moretti, F. Altermatt, B. Baur, and M.P. Nobis. 2016. Impacts of urban sprawl on species richness of plants, butterflies, gastropods and birds: Not only built-up area matters. *Urban Ecosystems* 19: 225–242. doi: 10.1007/s11252-015-0474-4

Drew, J.A., K.L. Amatangelo, and R.A. Hufbauer. 2015. Quantifying the human impacts on Papua New Guinea reef fish communities across space and time. *PloS One* 10: e0140682 (eng). doi: 10.1371/journal.pone.0140682

Edgar, G.J., and N.S. Barrett. 2000. Effects of catchment activities on macrofaunal assemblages in Tasmanian estuaries. *Estuarine, Coastal and Shelf Science* 50: 639–654. doi: 10.1006/ecss.2000.0591

Emmanuel, R. 1997. Urban vegetational change as an indicator of demographic trends in cities: The Case of Detroit. *Environment and Planning B: Planning and Design* 24: 415–426. doi: 10.1068/b240415

Engel, D.W., and G.W. Thayer. 1998. Effects of habitat alteration on blue crabs. *Journal of Shellfish Research* 17: 579–585.

Espinosa-García, F.J., J.L. Villaseñor, and H. Vibrans. 2004. The rich generally get richer, but there are exceptions: Correlations between species richness of native plant species and alien weeds in Mexico. *Diversity and Distributions* 10: 399–407. doi: 10.1111/j.1366-9516.2004.00099.x

Estes, A.B., T. Kuemmerle, H. Kushnir, V.C. Radeloff, and H.H. Shugart. 2012. Land-cover change and human population trends in the greater Serengeti ecosystem from 1984–2003. *Biological Conservation* 147: 255–263. doi: 10.1016/j.biocon.2012.01.010

Estrada, A. 2013. Socioeconomic contexts of primate conservation: population, poverty, global economic demands, and sustainable land use. *American Journal of Primatology* 75: 30–45 (eng). doi: 10.1002/ajp.22080

Evans, K.L., and K.J. Gaston. 2005. People, energy and avian species richness. *Global Ecology and Biogeography* 14: 187–196. doi: 10.1111/j.1466-822X.2004.00139.x

Evans, K.L., J.J.D. Greenwood, and K.J. Gaston. 2007. The positive correlation between avian species richness and human population density in Britain is not attributable to sampling bias. *Global Ecology and Biogeography* 16: 300–304. doi: 10.1111/j.1466-8238.2006.00288.x

Evans, K.L., B.J. van Rensburg, K.J. Gaston, and S.L. Chown. 2006. People, species richness and human population growth. *Global Ecology and Biogeography* 0: 060811081017002-??? doi: 10.1111/j.1466-822X.2006.00253.x

Evans, K.L., P.H. Warren, and K.J. Gaston. 2005. Does energy availability influence classical patterns of spatial variation in exotic species richness? *Global Ecology and Biogeography* 14: 57–65. doi: 10.1111/j.1466-822X.2004.00134.x

Figueroa, F., V. Sánche-Cordero, J. Meave, and I. Trejo. 2009. Socioeconomic context of land use and land cover change in Mexican biosphere reserves. *Environmental Conservation* 36: 180. doi: 10.1017/S0376892909990221

Fischer, L.K., V. Rodorff, M. von der Lippe, and I. Kowarik. 2016. Drivers of biodiversity patterns in parks of a growing South American megacity. *Urban Ecosystems* 19: 1231–1249. doi: 10.1007/s11252-016-0537-1

Fisher, B., and T. Christopher. 2006. Poverty and biodiversity: Measuring the overlap of human poverty and the biodiversity hotspots. *Ecological Economics* 62: 93–101. doi: 10.1016/j.ecolecon.2006.05.020

Fjeldså, J. 2007. The relationship between biodiversity and population centres: The high Andes region as an example. *Biodiversity and Conservation* 16: 2739–2751. doi: 10.1007/s10531-007-9204-4

Fjeldså, J., and N.D. Burgess. 2008. The coincidence of biodiversity patterns and human settlement in Africa. *African Journal of Ecology* 46: 33–42. doi: 10.1111/j.1365-2028.2008.00927.x

Foster, D.R., G. Motzkin, D. Bernardos, and J. Cardoza. 2002. Wildlife dynamics in the changing New England landscape. *Journal of Biogeography* 29: 1337–1357. doi: 10.1046/j.1365-2699.2002.00759.x

Gallant, A.L., R.W. Klaver, G.S. Casper, and M.J. Lannoo. 2007. Global rates of habitat loss and implications for amphibian conservation. *Copeia* 2007: 967–979. doi: 10.1643/0045-8511(2007)7[967:GROHLA]2.0.CO;2

Gibbs, J.P. 2000. Wetland loss and biodiversity conservation. *Conservation Biology* 14: 314–317. doi: 10.1046/j.1523-1739.2000.98608.x

Grau, H.R., N.I. Gasparri, and T.M. Aide. 2008. Balancing food production and nature conservation in the Neotropical dry forests of northern Argentina. *Global Change Biology* 14: 985–997. doi: 10.1111/j.1365-2486.2008.01554.x

Gupta, A.C. 2015. Spatial scaling of protected area influences on human demography and livelihoods in Botswana. *Environmental Conservation* 42: 51–60. doi: 10.1017/S0376892914000095

Habel, J.C., M. Teucher, R.K. Mulwa, W. Haber, H. Eggermont, and L. Lens. 2016. Nature conservation at the edge. *Biodiversity and Conservation* 25: 791–799. doi: 10.1007/s10531-016-1073-2

Hansen, A.J., R. Rasker, B. Maxwell, J.J. Rotella, J.D. Johnson, A.W. Parmenter, U. Langner, W.B. Cohen, et al. 2002. Ecological causes and consequences of demographic change in the New West. *BioScience* 52: 151. doi: 10.1641/0006-3568(2002)052[0151:ECACOD]2.0.CO;2

Hanspach, J., J. Loos, I. Dorresteijn, D.J. Abson, J. Fischer, and E. Di Minin. 2016. Characterizing social-ecological units to inform biodiversity conservation in cultural landscapes. *Diversity and Distributions* 22: 853–864. doi: 10.1111/ddi.12449

Harris, A., V. Mohan, M. Flanagan, and R. Hill. 2012. Integrating family planning service provision into community-based marine conservation. *Oryx* 46: 179–186. doi: 10.1017/S0030605311000925

Hartter, J., S.J. Ryan, C.A. MacKenzie, A. Goldman, N. Dowhaniuk, M. Palace, J.E. Diem, and C.A. Chapman. 2015. Now there is no land: A story of ethnic migration in a protected area landscape in western Uganda. *Population and Environment* 36: 452–479. doi: 10.1007/s11111-014-0227-y

Hartter, J., and J. Southworth. 2009. Dwindling resources and fragmentation of landscapes around parks: Wetlands and forest patches around Kibale National Park, Uganda. *Landscape Ecology* 24: 643–656. doi: 10.1007/s10980-009-9339-7

Hiller, T.L., J.E. Mcfadden-Hiller, S.R. Jenkins, J.L. Belant, and A.J. Tyre. 2015. Demography, prey abundance, and management affect number of cougar mortalities associated with livestock conflicts. *The Journal of Wildlife Management* 79: 978–988. doi: 10.1002/jwmg.913

Holland, A.F., D.M. Sanger, C.P. Gawle, S.B. Lerberg, M.S. Santiago, G.H.M. Riekerk, L.E. Zimmerman, and G.I. Scott. 2004. Linkages between tidal creek ecosystems and the landscape and demographic attributes of their watersheds. *Journal of Experimental Marine Biology and Ecology* 298: 151–178. doi: 10.1016/S0022-0981(03)00357-5

Hugo, S., and B.J. van Rensburg. 2008. The maintenance of a positive spatial correlation between South African bird species richness and human population density. *Global Ecology and Biogeography* 17: 611–621. doi: 10.1111/j.1466-8238.2008.00391.x

Hunter, L.M., M.J. de Gonzalez G, M. Stevenson, K.S. Karish, R. Toth, T.C. Edwards, Jr., R.J. Lilieholm, and M. Cablk. 2003. Population and land use change in the California Mojave: Natural habitat implications of alternative futures. *Population Research and Policy Review* 22: 373–397. doi: 10.1023/A:1027311225410

Huston, M.A. 2005. The three phases of land-use change: Implications for biodiversity. *Ecological Applications* 15: 1864–1878.

Inogwabini, B.-I. 2014. Bushmeat, over-fishing and covariates explaining fish abundance declines in the Central Congo Basin. *Environmental Biology of Fishes* 97: 787–796. doi: 10.1007/s10641-013-0179-6

Iwamura, T., E.F. Lambin, K.M. Silvius, J.B. Luzar, and J.M.V. Fragoso. 2014. Agent-based modeling of hunting and subsistence agriculture on indigenous lands: Understanding interactions between social and ecological systems. *Environmental Modelling & Software* 58: 109–127. doi: 10.1016/j.envsoft.2014.03.008

Izazola, H., C. Martínez, and C. Marquette. 1998. Evironmental perceptions, social class and demographic change in Mexico City: A comparative approach. *Environment and Urbanization* 10: 107–118. doi: 10.1177/095624789801000101

Izquierdo, A.E., C.D. De Angelo, and T.M. Aide. 2008. Thirty years of human demography and land-use change in the Atlantic forest of Missiones, Argentina: An evaluation of the forest transition model. *Ecology and Society* 13: 3.

Izquierdo, A.E., and H.R. Grau. 2009. Agriculture adjustment, land-use transition and protected areas in Northwestern Argentina. *Journal of Environmental Management* 90: 858–865 (eng). doi: 10.1016/j.jenvman.2008.02.013

Jha, S., and K.S. Bawa. 2006. Population growth, human development, and deforestation in biodiversity hotspots. *Conservation Biology* 20: 906–912. doi: 10.1111/j.1523-1739.2006.00398.x

Joppa, L.N., S.R. Loarie, and S.L. Pimm. 2009. On population growth near protected areas. *PloS One* 4: e4279 (eng). doi: 10.1371/journal.pone.0004279

Kampichler, C., S. Calm, H. Weissenberger, and S.L. Arriaga-Weiss. 2010. Indication of a species in an extinction vortex: The ocellated turkey on the Yucatan peninsula, Mexico. *Acta Oecologica* 36: 561–568. doi: 10.1016/j.actao.2010.08.004

Keith, M., and M. Warren. 2007. Can anthropogenic variables be used as threat proxies for South African plant richness? *Bothalia* 37: 79–88. doi: 10.4102/abc.v37i1.305

Kerr, J.T., and T.V. Burkey. 2002. Endemism, diversity, and the threat of tropical moist forest extinctions. *Biodiversity and Conservation* 11: 695–704. doi: 10.1023/A:1015500513603

Koenig, W.D., W.M. Hochachka, B. Zuckerberg, and J.L. Dickinson. 2010. Ecological determinants of American crow mortality due to West Nile virus during its North American sweep. *Oecologia* 163: 903–909 (eng). doi: 10.1007/s00442-010-1627-z

Koh, C.-N., P.-F. Lee, and R.-S. Lin. 2006. Bird species richness patterns of northern Taiwan: Primary productivity, human population density, and habitat heterogeneity. *Diversity and Distributions* 12: 546–554. doi: 10.1111/j.1366-9516.2006.00238.x
Laundré, J.W., J.L. Salazar, L. Hernández, and D.N. López. 2009. Evaluating potential factors affecting puma *Puma concolor* abundance in the Mexican Chihuahuan Desert. *Wildlife Biology* 15: 207–212. doi: 10.2981/07-077
Laurance, W.F. 2007. Have we overstated the tropical biodiversity crisis? *Trends in Ecology & Evolution* 22: 65–70 (eng). doi: 10.1016/j.tree.2006.09.014
Lepczyk, C.A., C.H. Flather, V.C. Radeloff, A.M. Pidgeon, R.B. Hammer, and J. Liu. 2008. Human impacts on regional avian diversity and abundance. *Conservation biology : the journal of the Society for Conservation Biology* 22: 405–416 (eng). doi: 10.1111/j.1523-1739.2008.00881.x
Levi, T., G.H. Shepard Jr, J. Ohl-Schacherer, C.A. Peres, and D.W. Yu. 2009. Modelling the long-term sustainability of indigenous hunting in Manu National Park, Peru: Landscape-scale management implications for Amazonia. *Journal of Applied Ecology* 46: 804–814. doi: 10.1111/j.1365-2664.2009.01661.x
Liu, J., G.C. Daily, P.R. Ehrlich, and G.W. Luck. 2003. Effects of household dynamics on resource consumption and biodiversity. *Nature* 421: 530–533 (eng). doi: 10.1038/nature01359
Liu, J., Z. Ouyang, Y. Tan, J. Yang, and H. Zhang. 1999. Changes in human population structure: Implications for biodiversity conservation. *Population and Environment* 21: 45–58. doi: 10.1007/BF02436120
Liu, J., and A. Viña. 2014. Pandas, plants, and people. *Annals of the Missouri Botanical Garden* 100: 108–125. doi: 10.3417/2013040
López-Bao, J.V., J.P. González-Varo, and J. Guitián. 2015. Mutualistic relationships under landscape change: Carnivorous mammals and plants after 30 years of land abandonment. *Basic and Applied Ecology* 16: 152–161. doi: 10.1016/j.baae.2014.12.001
López-Medellín, X., A.G. Navarro-Sigüenza, and G. Bocco. 2011. Human population, economic activities, and wild bird conservation in Mexico: factors influencing their relationships at two different geopolitical scales. *Revista Mexicana de Biodiversidad* 82: 1267–1278.
Lozano, J., and A.F. Malo. 2013. Relationships between human activity and richness and abundance of some bird species in the Paraguay River (Pantanal, Brazil). *Ardeola* 60: 99–112. doi: 10.13157/arla.60.1.2012.99
Luck, G.W. 2007. The relationships between net primary productivity, human population density and species conservation. *Journal of Biogeography* 34: 201–212. doi: 10.1111/j.1365-2699.2006.01575.x
Luck, G.W., T.H. Ricketts, G.C. Daily, and M. Imhoff. 2004. Alleviating spatial conflict between people and biodiversity. *Proceedings of the National Academy of Sciences of the United States of America* 101: 182–186 (eng). doi: 10.1073/pnas.2237148100
Luck, G.W., L. Smallbone, S. McDonald, and D. Duffy. 2010. What drives the positive correlation between human population density and bird species richness in Australia? *Global Ecology and Biogeography* 12: no-no. doi: 10.1111/j.1466-8238.2010.00545.x
Mammides, C., C. Kadis, and T. Coulson. 2015. The effects of road networks and habitat heterogeneity on the species richness of birds in Natura 2000 sites in Cyprus. *Landscape Ecology* 30: 67–75. doi: 10.1007/s10980-014-0100-5
Marini, L., K.J. Gaston, F. Prosser, and P.E. Hulme. 2009. Contrasting response of native and alien plant species richness to environmental energy and human impact along alpine elevation gradients. *Global Ecology and Biogeography* 18: 652–661. doi: 10.1111/j.1466-8238.2009.00484.x
Maurer, B.A. 1996. Relating human population growth to the loss of biodiversity. *Biodiversity Letters* 3: 1–5. doi: 10.2307/2999702
McKee, J.K., P.W. Sciulli, C.D. Fooce, and T.A. Waite. 2003. Forecasting global biodiversity threats associated with human population growth. *Biological Conservation* 115: 161–164. doi: 10.1016/S0006-3207(03)00099-5
McKinney, M.L. 2002. Urbanization, biodiversity, and conservation. *BioScience* 52: 883–890. doi: 10.1641/0006-3568(2002)052[0883:UBAC]2.0.CO;2
McMullin, R.T., L.L. Bennet, O.J. Bjorgan, D.A. Bourque, C.J. Burke, M.A. Clarke, M.K. Gutgesell, P.L. Krawiec, et al. 2016. Relationships between air pollution, population density, and lichen biodiversity in the Niagara Escarpment World Biosphere Reserve. *The Lichenologist* 48: 593–605. doi: 10.1017/S0024282916000402
McSweeney, K. 2005. Indigenous population growth in the lowland neotropics: Social science insights for biodiversity conservation. *Conservation Biology* 19: 1375–1384. doi: 10.1111/j.1523-1739.2005.00194.x
Mecenero, S., R. Altwegg, J.F. Colville, and C.M. Beale. 2015. Roles of spatial scale and rarity on the relationship between butterfly species richness and human density in South Africa. *PloS One* 10: e0124327 (eng). doi: 10.1371/journal.pone.0124327
Metzger, K.L., A.R.E. Sinclair, R. Hilborn, J.G.C. Hopcraft, and S.A.R. Mduma. 2010. Evaluating the protection of wildlife in parks: The case of African buffalo in Serengeti. *Biodiversity and Conservation* 19: 3431–3444. doi: 10.1007/s10531-010-9904-z
Mora, C., O. Aburto-Oropeza, A. Ayala Bocos, P.M. Ayotte, S. Banks, A.G. Bauman, M. Beger, S. Bessudo, et al. 2011. Global human footprint on the linkage between biodiversity and ecosystem functioning in reef fishes. *PLoS Biology* 9: e1000606 (eng). doi: 10.1371/journal.pbio.1000606
Nanni, A.S., and H.R. Grau. 2014. Agricultural adjustment, population dynamics and forests redistribution in a subtropical watershed of NW Argentina. *Regional Environmental Change* 14: 1641–1649. doi: 10.1007/s10113-014-0608-x
Newbold, T., L.N. Hudson, H.R.P. Phillips, S.L.L. Hill, S. Contu, I. Lysenko, A. Blandon, S.H.M. Butchart, et al. 2014. A global model of the response of tropical and sub-tropical forest biodiversity to anthropogenic pressures. *Proceedings of the Royal Society B* 281 (eng). doi: 10.1098/rspb.2014.1371
Olden, J.D., N.L. Poff, and M.L. McKinney. 2006. Forecasting faunal and floral homogenization associated with human population geography in North America. *Biological Conservation* 127: 261–271. doi: 10.1016/j.biocon.2005.04.027
Paudel, P.K., P. Kindlmann, I. Gordon, and C. Mishra. 2012. Human disturbance is a major determinant of wildlife distribution in Himalayan midhill landscapes of Nepal. *Animal Conservation* 15: 283–293. doi: 10.1111/j.1469-1795.2011.00514.x
Pautasso, M. 2007. Scale dependence of the correlation between human population presence and vertebrate and plant species richness. *Ecology Letters* 10: 16–24 (eng). doi: 10.1111/j.1461-0248.2006.00993.x
Pautasso, M., and A. Chiarucci. 2008. A test of the scale-dependence of the species abundance-people correlation for veteran trees in Italy. *Annals of Botany* 101: 709–715 (eng). doi: 10.1093/aob/mcn010
Pautasso, M., and M. Dinetti. 2009. Avian species richness, human population and protected areas across Italy's regions. *Environmental Conservation* 36: 22. doi: 10.1017/S037689290900544X
Pautasso, M., and D. Fontaneto. 2008. A test of the species-people correlation for stream macro-invertebrates in European countries. *Ecological Applications* 18: 1842–1849 (eng).
Pautasso, M., and G. Powell. 2009. Aphid biodiversity is positively correlated with human population in European countries. *Oecologia* 160: 839–846 (eng). doi: 10.1007/s00442-009-1329-6
Pecher, C., S.A. Fritz, L. Marini, D. Fontaneto, and M. Pautasso. 2010. Scale-dependence of the correlation between human population and the species richness of stream macro-invertebrates. *Basic and Applied Ecology* 11: 272–280. doi: 10.1016/j.baae.2009.09.005
Pinto, R., V.N. de Jonge, J.M. Neto, T. Domingos, J.C. Marques, and J. Patrício. 2013. Towards a DPSIR driven integration of ecological value, water uses and ecosystem services for estuarine systems. *Ocean & Coastal Management* 72: 64–79. doi: 10.1016/j.ocecoaman.2011.06.016
Poulsen, J.R., C.J. Clark, G. Mavah, and P.W. Elkan. 2009. Bushmeat supply and consumption in a tropical logging concession in northern Congo. *Conservation biology : the journal of the Society for Conservation Biology* 23: 1597–1608 (eng). doi: 10.1111/j.1523-1739.2009.01251.x
Prado, H.M., L.C. Forline, and R. Kipnis. 2012. Hunting practices among the Awá-Guajá: towards a long-term analysis of sustainability in an Amazonian indigenous community. *Boletim do Museu Paraense Emílio Goeldi Ciências Humanas* 7: 479–491. doi: 10.1590/S1981-81222012000200010

Pysek, P., V. Jarosík, P.E. Hulme, I. Kühn, J. Wild, M. Arianoutsou, S. Bacher, F. Chiron, et al. 2010. Disentangling the role of environmental and human pressures on biological invasions across Europe. *Proceedings of the National Academy of Sciences of the United States of America* 107: 12157–12162 (eng). doi: 10.1073/pnas.1002314107

Quan, R.-C., Y. Huang, M.W. Warren, Q.-K. Zhao, G. Ren, S. Huo, Y. Long, and J. Zhu. 2011. How human household size affects the habitat of black-and-white snub-nosed monkeys (Rhinopithecus bieti) in Hongla Snow Mountain Nature Reserve in Tibet, China. *International Journal of Primatology* 32: 1190–1202. doi: 10.1007/s10764-011-9535-6

Radeloff, V.C., R.B. Hammer, P.R. Voss, A.E. Hagen, D.R. Field, and D.J. Mladenoff. 2001. Human demographic trends and landscape level forest management in the Northwest Wisconsin Pine Barrens. *Forest Science* 47: 229–241.

Ramos, W.R., J.F. Medeiros, G.R. Julião, C.M. Ríos-Velásquez, E.F. Marialva, S.J.M. Desmouliére, S.L.B. Luz, and F.A.C. Pessoa. 2014. Anthropic effects on sand fly (Diptera Psychodidae) abundance and diversity in an Amazonian rural settlement, Brazil. *Acta tropica* 139: 44–52 (eng). doi: 10.1016/j.actatropica.2014.06.017

Rangel, T.F.L.V.B., L.M. Bini, J.A.F. Diniz-Filho, M.P. Pinto, P. Carvalho, and R.P. Bastos. 2007. Human development and biodiversity conservation in Brazilian Cerrado. *Applied Geography* 27: 14–27. doi: 10.1016/j.apgeog.2006.09.009

Reid, R.S., R.L. Kruska, U. Deichmann, P.K. Thornton, and S.G.A. Leak. 2000. Human population growth and the extinction of the tsetse fly. *Agriculture, Ecosystems & Environment* 77: 227–236. doi: 10.1016/S0167-8809(99)00103-6

Remis, M.J., and J.B. Kpanou. 2011. Primate and ungulate abundance in response to multi-use zoning and human extractive activities in a Central African Reserve. *African Journal of Ecology* 49: 70–80. doi: 10.1111/j.1365-2028.2010.01229.x

Restrepo, A., V.P. Páez, C. López, and B.C. Bock. 2008. Distribution and status of podocnemis lewyana in the Magdalena River drainage of Colombia. *Chelonian Conservation and Biology* 7: 45–51. doi: 10.2744/CCB-0668.1

Richards, B.L., I.D. Williams, O.J. Vetter, and G.J. Williams. 2012. Environmental factors affecting large-bodied coral reef fish assemblages in the Mariana Archipelago. *PloS One* 7: e31374 (eng). doi: 10.1371/journal.pone.0031374

Robichaud, W.G., A.R.E. Sinclair, N. Odarkor-Lanquaye, and B. Klinkenberg. 2009. Stable forest cover under increasing populations of swidden cultivators in central Laos: the roles of intrinsic culture and extrinsic wildlife trade. *Ecology and Society* 14: 1.

Rochlin, I., K. Harding, H.S. Ginsberg, and S.R. Campbell. 2008. Comparative analysis of distribution and abundance of West Nile and eastern equine encephalomyelitis virus vectors in Suffolk County, New York, using human population density and land use/cover data. *Journal of Medical Entomology* 45: 563–571 (eng).

Rompré, G., W.D. Robinson, and A. Desrochers. 2008. Causes of habitat loss in a Neotropical landscape: The Panama Canal corridor. *Landscape and Urban Planning* 87: 129–139. doi: 10.1016/j.landurbplan.2008.05.006

Saqib, Z., R.N. Malik, and H. von Wehrden. 2013. Landcover dynamics in relation to Western Tragopan occurrence in Pakistan: a regional assessment. *Pakistan Journal of Botany* 45: 551–559.

Schlick-Steiner, B.C., F.M. Steiner, and M. Pautasso. 2008. Ants and people: A test of two mechanisms potentially responsible for the large-scale human population-biodiversity correlation for Formicidae in Europe. *Journal of Biogeography* 35: 2195–2206. doi: 10.1111/j.1365-2699.2008.01968.x

Selier, S.-A.J., R. Slotow, and E. Di Minin. 2016. The influence of socioeconomic factors on the densities of high-value cross-border species, the African elephant. *PeerJ* 4: e2581. doi: 10.7717/peerj.2581

Spear, D., L.C. Foxcroft, H. Bezuidenhout, and M.A. McGeoch. 2013. Human population density explains alien species richness in protected areas. *Biological Conservation* 159: 137–147. doi: 10.1016/j.biocon.2012.11.022

Steck, C.E., and M. Pautasso. 2008. Human population, grasshopper and plant species richness in European countries. *Acta Oecologica* 34: 303–310. doi: 10.1016/j.actao.2008.06.003

Stoner, E.W., C.A. Layman, L.A. Yeager, and H.M. Hassett. 2011. Effects of anthropogenic disturbance on the abundance and size of epibenthic jellyfish Cassiopea spp. *Marine Pollution Bulletin* 62: 1109–1114 (eng). doi: 10.1016/j.marpolbul.2011.03.023

Svancara, L.K., J.M. Scott, T.R. Loveland, and A.B. Pidgorna. 2009. Assessing the landscape context and conversion risk of protected areas using satellite data products. *Remote Sensing of Environment* 113: 1357–1369. doi: 10.1016/j.rse.2008.11.015

Swierk, L., and S.R. Madigosky. 2014. Environmental perceptions and resource use in rural communities of the Peruvian Amazon (Iquitos and Vicinity, Maynas Province). *Tropical Conservation Science* 7: 382–402. doi: 10.1177/194008291400700303

Tavernia, B.G., M.D. Nelson, P. Caldwell, and G. Sun. 2013. Water stress projections for the northeastern and midwestern United States in 2060: Anthropogenic and Ecological Consequences. *JAWRA Journal of the American Water Resources Association* 49: 938–952. doi: 10.1111/jawr.12075

Terer, T., A.M. Muasya, F. Dahdouh-Guebas, G.G. Ndiritu, and L. Triest. 2012. Integrating local ecological knowledge and management practices of an isolated semi-arid papyrus swamp (Loboi, Kenya) into a wider conservation framework. *Journal of Environmental Management* 93: 71–84 (eng). doi: 10.1016/j.jenvman.2011.08.005

Thoisy, B. de, C. Richard-Hansen, B. Goguillon, P. Joubert, J. Obstancias, P. Winterton, and S. Brosse. 2010. Rapid evaluation of threats to biodiversity: Human footprint score and large vertebrate species responses in French Guiana. *Biodiversity and Conservation* 19: 1567–1584. doi: 10.1007/s10531-010-9787-z

Trawinski, P.R., and D.S. Mackay. 2010. Identification of environmental covariates of West Nile virus vector mosquito population abundance. *Vector Borne and Zoonotic Diseases* 10: 515–526 (eng). doi: 10.1089/vbz.2008.0063

Underwood, E.C., J.H. Viers, K.R. Klausmeyer, R.L. Cox, and M.R. Shaw. 2009. Threats and biodiversity in the mediterranean biome. *Diversity and Distributions* 15: 188–197. doi: 10.1111/j.1472-4642.2008.00518.x

Urquiza-Haas, T., C.A. Peres, and P.M. Dolman. 2009. Regional scale effects of human density and forest disturbance on large-bodied vertebrates throughout the Yucatán Peninsula, Mexico. *Biological Conservation* 142: 134–148. doi: 10.1016/j.biocon.2008.10.007

Vačkář, D., K. Chobot, and E. Orlitová. 2012. Spatial relationship between human population density, land use intensity and biodiversity in the Czech Republic. *Landscape Ecology* 27: 1279–1290. doi: 10.1007/s10980-012-9779-3

Vimal, R., P. Pluvinet, C. Sacca, P.-O. Mazagol, B. Etlicher, and J.D. Thompson. 2012. Exploring spatial patterns of vulnerability for diverse biodiversity descriptors in regional conservation planning. *Journal of Environmental Management* 95: 9–16 (eng). doi: 10.1016/j.jenvman.2011.09.018

Walker, K.R., T.K. Joy, C. Ellers-Kirk, and F.B. Ramberg. 2011. Human and environmental factors affecting Aedes aegypti distribution in an arid urban environment. *Journal of the American Mosquito Control Association* 27: 135–141 (eng). doi: 10.2987/10-6078.1

White, P., and J.T. Kerr. 2006. Contrasting spatial and temporal global change impacts on butterfly species richness during the 20th century. *Ecography* 29: 908–918. doi: 10.1111/j.2006.0906-7590.04685.x

Williams, I.D., J.K. Baum, A. Heenan, K.M. Hanson, M.O. Nadon, and R.E. Brainard. 2015. Human, oceanographic and habitat drivers of central and western Pacific coral reef fish assemblages. *PloS One* 10: e0120516 (eng). doi: 10.1371/journal.pone.0120516

Williams, I.D., W.J. Walsh, R.E. Schroeder, A.M. Friedlander, B.L. Richards, and K.A. Stamoulis. 2008. Assessing the importance of fishing impacts on Hawaiian coral reef fish assemblages along regional-scale human population gradients. *Environmental Conservation* 35: 261. doi: 10.1017/S0376892908004876

Wilson, J.W., B.J. van Rensburg, J.W.H. Ferguson, and M. Keith. 2007. The relative importance of environment, human activity and space in explaining species richness of South African bird orders. *Journal of Biogeography* 0: 071103055558001-??? doi: 10.1111/j.1365-2699.2007.01792.x

Wittemyer, G., P. Elsen, W.T. Bean, A.C.O. Burton, and J.S. Brashares. 2008. Accelerated human population growth at protected area edges. *Science* 321: 123–126 (eng). doi: 10.1126/science.1158900

Wright, S.J., and H.C. Muller-Landau. 2006. The future of tropical forest species. *Biotropica* 38: 287–301. doi: 10.1111/j.1744-7429.2006.00154.x

Zaidi, F., S.H. Fatima, M. Khisroon, and A. Gul. 2016. Distribution modeling of three screwworm species in the ecologically diverse landscape of North West Pakistan. *Acta tropica* 162: 56–65 (eng). doi: 10.1016/j.actatropica.2016.06.015

Zhao, S., J. Fang, S. Miao, B. Gu, S. Tao, C. Peng, and Z. Tang. 2005. The 7-decade degradation of a large freshwater lake in central Yangtze River, China. *Environmental Science & Technology* 39: 431–436 (eng).

Zick, D., H. Gassner, P. Filzmoser, J. Wanzenböck, B. Pamminger-Lahnsteiner, and G. Tischler. 2006. Changes in the fish species composition of all Austrian lakes >50 ha during the last 150 years. *Fisheries Management and Ecology* 13: 103–111. doi: 10.1111/j.1365-2400.2006.00483.x
